# Insights From Genetic Studies of Cerebral Palsy

**DOI:** 10.3389/fneur.2020.625428

**Published:** 2021-01-21

**Authors:** Sara A. Lewis, Sheetal Shetty, Bryce A. Wilson, Aris J. Huang, Sheng Chih Jin, Hayley Smithers-Sheedy, Michael C. Fahey, Michael C. Kruer

**Affiliations:** ^1^Pediatric Movement Disorders Program, Barrow Neurological Institute, Phoenix Children's Hospital, Phoenix, AZ, United States; ^2^Departments of Child Health, Neurology, and Cellular & Molecular Medicine and Program in Genetics, University of Arizona College of Medicine, Phoenix, AZ, United States; ^3^Programs in Neuroscience and Molecular & Cellular Biology, School of Life Sciences, Arizona State University, Tempe, AZ, United States; ^4^Department of Genetics, Washington University School of Medicine, St. Louis, MO, United States; ^5^Cerebral Palsy Alliance, Sydney Medical School, The University of Sydney, Sydney, NSW, Australia; ^6^Department of Paediatrics, Monash University, Melbourne, VIC, Australia

**Keywords:** cerebral palsy, genetics, genomics, neurodevelopmental disorders, neurogenetics

## Abstract

Cohort-based whole exome and whole genome sequencing and copy number variant (CNV) studies have identified genetic etiologies for a sizable proportion of patients with cerebral palsy (CP). These findings indicate that genetic mutations collectively comprise an important cause of CP. We review findings in CP genomics and propose criteria for CP-associated genes at the level of gene discovery, research study, and clinical application. We review the published literature and report 18 genes and 5 CNVs from genomics studies with strong evidence of for the pathophysiology of CP. CP-associated genes often disrupt early brain developmental programming or predispose individuals to known environmental risk factors. We discuss the overlap of CP-associated genes with other neurodevelopmental disorders and related movement disorders. We revisit diagnostic criteria for CP and discuss how identification of genetic etiologies does not preclude CP as an appropriate diagnosis. The identification of genetic etiologies improves our understanding of the neurobiology of CP, providing opportunities to study CP pathogenesis and develop mechanism-based interventions.

## Introduction

Cerebral palsy (CP) describes a disorder of motor function resulting from maldevelopment or injury to the developing brain. The motor disorders of CP are frequently accompanied by other associated impairments including intellectual disability, epilepsy and sensory impairments (1). CP is relatively common with an estimated prevalence of 1.3–1.9 cases per 1,000 live births in high income countries (2, 3). Whilst often described as a childhood disorder, CP is a lifelong condition. Although many risk factors have been recognized, for many individuals identifying the etiology of their CP can be difficult.

One example of the complexity of CP etiology is preterm birth. Babies born preterm have an increased risk of CP. However, not all babies born preterm go on to develop CP. Being born preterm puts an infant at-risk for additional complications, including respiratory injury (hypoxia, hypercarbia, or hyperoxia), infectious/inflammatory insults (sepsis or necrotizing enterocolitis), hemodynamic compromise (hypotension, intraventricular hemorrhage, or thrombosis). These factors can contribute to cell death in the brain or alter the maturation of neurons and glia, resulting in abnormal white matter tracts (4) diminished cerebral volumes (5), or cerebellar hypoplasia (6). Further, genetic factors contribute to risk for preterm birth (7). Together this indicates the pathophysiology of CP may result from a combination of genetic, environmental, and genetic factors. Other CP risk factors, such as congenital anomalies or intrauterine growth restriction, may also reflect underlying genetic etiologies (8–11). Recognizing how genetic changes contribute to CP is a rapidly developing field with important themes.

## Genetic Etiologies of CP

### Relative Contribution of Copy Number Variants

Recent studies have implicated genetic factors as contributors or causes of CP, including single nucleotide variants (SNVs) and genomic copy number variants (CNVs). Putatively deleterious CNVs have been found in several CP cohorts, although estimates of molecular etiologic yield have varied considerably depending on study criteria (12–16). The initial study of 50 unselected CP cases using microarrays implicated novel variants in CP (12). Segel et al. (13) used microarrays to study an enriched cohort of 52 individuals with cryptogenic CP (i.e., no known cause). By applying American College of Medical Genetics & Genomics (ACMG) criteria to interpret their results, they identified 31% with pathogenic or likely pathogenic CNVs. Oskoui et al. (14) studied an unselected cohort of 147 individuals with CP using microarrays and evaluated detected CNVs using rigorous criteria based on ACMG guidelines. They found evidence for pathogenic or likely pathogenic CNVs in 9.6% of their cases. Zarrei et al. (15) assessed pathogenic or likely pathogenic CNVs in 97 individuals with otherwise unselected hemiplegic CP. When focusing on rare, *de novo* CNVs, they identified a molecular diagnostic yield of 7.2%. When they expanded their criteria to include inherited CNVs, known DECIPHER or ClinGen loci or CNVs that encompass genes known to play a role in NDD or brain/muscle disease, the potential yield rose to 23.7%. Finally, Corbett et al. (16) assessed CNV determination from whole exome data, finding an additional 3.7% of cases could be considered “solved” when CNVs are considered alongside SNVs.

In these studies, recurrent likely pathogenic CNVs were identified, such as a 2p25.3 deletion, 22q11.2 deletions and duplications, and Xp monosomy (**Table 2**). The relationship between a genetic deletion/duplication and CP can be complex. For example, 22q11.2 deletions can be associated with major congenital heart defects and polymicrogyria, with the cardiac abnormalities predisposing individuals to stroke and perioperative complications. Alternatively, 22q11.2 deletions are associated with dystonia in the absence of cardiac anomalies (17, 18). In addition, efforts to identify pathogenic CNVs are limited by clinical variability and microdeletion/microduplication boundaries that only partially overlap. Continued analysis and reporting of CNV findings alongside massively parallel SNV analyses are needed to expand knowledge of CNVs that can lead to CP.

### Relative Contribution of SNVs

The foundational study conducted by McMichael et al. (10) sequenced 98 unselected CP trios and found that six (6%) had a *de novo* (4 cases) or inherited (2 cases) predicted deleterious variant in a known disease-associated gene. Eight (8%) harbored a potentially pathogenic variant in a novel candidate CP gene. Six of these were *de novo* and two were inherited. A small study conducted by Schnekenberg et al. (19) found *de novo* mutations by WES in 3 out of 4 cases with ataxic CP. Subsequently, Takezawa et al. (20) whole exome sequenced 17 CP trios. These cases represented full-term births without diagnostic MRI findings. Nine of the 17 (53%) cases had pathogenic or likely pathogenic variants in known disease-associated genes by ACMG criteria.

Although these initial studies laid the foundation for the field, there were challenges in reconciling findings in candidate genes with known Online Mendelian Inheritance in Man (OMIM; https://www.omim.org) genes. Limitations of these studies included small sample sizes and a lack of robust statistical methods and well-matched controls. An international collaborative publication (21) addressed several of these limitations, reporting the largest trio-based cohort to date using a rigorously vetted statistical approach supported by mechanistic and biological validations.

Advanced paternal age had previously been associated with risk for CP (22) and is known to be associated with *de novo* mutations. *De novo* mutations were found to be enriched 1.2-fold in CP cases compared to the expected value estimated from background germline mutation rate (21, 23). Models predict between 27 and 124 genes likely contribute to CP through a *de novo* mechanism (21), with the number of identified genes therefore anticipated to rise exponentially with the number of trios sequenced. Gene discovery for CP is a growing area of interest with a recent literature search reporting 57 published genetic studies on individuals described as having CP. However, many of these reports are case studies and some included cases which do not meet current consensus criteria for CP (24), complicating interpretation.

With the increase in our knowledge of the role that genetics play in CP, distinctions between genetic and environmental/acquired etiologies are blurring and mixed and multiple insults are increasingly recognized. In some cases, genetic risk factors predispose individuals to environmental insults. Such a predisposition has been described for hemorrhagic stroke due to *COL4A1* and *COL4A2* mutations (25) and hypotonia before birth causing delivery complications and perinatal asphyxia, exemplified by 1p36 deletion syndrome (26). Differences in genetic backgrounds, such as single nucleotide polymorphisms in glutamate transport (27) and COX-1/2 receptors (28), might also underly susceptibility to CP after exposure to environmental risk factors (29).

The long-recognized increased risk of CP for males (30) also provides evidence for complex interactions between genetic contributions, predisposition, and responses to brain injury. For instance, female rats have more dystonia-like outcomes, and males have more spasticity-like outcomes after the same hypoxic-ischemic injury (31). Together, this suggests differences in genetics and subsequent physiology likely play a role in determining the development of CP in individuals exposed to environmental risk factors. Despite the considerable advances that have been made, the relative contribution of environmental vs. genetic causal factors remains unclear.

## What Constitutes a CP-Associated Gene?

Using careful case ascertainment to ensure sequenced individuals have CP is crucial for interpretation of genetic findings as either consistent with CP or not (24). Some cohorts may include individuals with progressive neurological impairments who do not fulfill the criteria for CP (32). Although these findings are important in their own right, genetic findings from individuals with rare pediatric movement disorders that do not meet the diagnostic criteria for CP should not be included in lists of CP-associated genes. Full reporting of the age of onset, movement disorder subtype, the presence or absence of neurodegeneration (including if there was follow-up over time and age of the last contact) and comorbidities will be necessary for the interpretation of the pathogenicity of variants in future cases presenting with mutations in those genes.

Determining whether a genetic variant or group of variants accounts for an individual's phenotype can be difficult without subsequent laboratory-based validation studies delineating the variant's effect on RNA or protein function. In some instances, the detection of multiple individuals in a CP cohort with predicted damaging variants in the same gene can provide strong statistical evidence for a *bona fide* association with CP if enrichment is present and consistent inheritance patterns are evident. A few CP-associated genes have clear loss-of-function variants, such as stop gain and frameshift variants in *CTNNB1* (21). Splice site variants can be challenging to interpret, but have been confirmed to affect mRNA sequence for *AMPD2* and *CTNNB1* as well as protein function in the case of *AMPD2* (20). However, most variants are missense mutations, and when bioinformatically predicted deleterious *de novo* missense mutations are systematically tested, many do not change protein function (33), highlighting the importance of experimental validation. *De novo* variants also can also contribute through gain of function or even change of function effects. For example, *FBXO31* and *RHOB* variants did not demonstrate loss of function deficits. However, when studied in more detail, gain/change of function mechanisms were identified (21). Given that many genes can have multiple functions, diverse localizations and myriad interactions, defects can be hard to detect in a single assay, making it more challenging to rule out candidate genes as well. *TUBA1A* missense variants, for example, can have subtle effects on microtubule shape and protein interactions, which may contribute to considerable phenotype heterogeneity (34). Even in some cases where genes have been shown to have an essential role in movement in model organism studies using zebrafish or Drosophila, the human variants were not tested to confirm variant-specific effects (21, 35).

The sheer number of genes that are being implicated in CP translates into a small but growing number of recurrent genes with a limited number of affected patients harboring variants in these genes. Larger cohorts with cases and controls utilizing methods to detect statistical enrichment of genes with deleterious variants are needed to overcome the recurrence gap. Gene matching platforms such as GeneMatcher (36) are going to be crucial for finding other patients with these rare genetic variants with clinical concordance. Current approaches to overcome limitations in identifying recurrent genes include using pathway enrichment and disease gene overlap to identify genes with a higher probability of contributing to CP pathology.

The criteria for identifying “a CP-associated gene” may vary based on the context prompting the question. Determining whether genetic variants explain an individual's clinical condition requires more stringent criteria than may be required for studying genetic networks contributing to the disease through multifactorial mechanisms. We propose a list of criteria for evaluating the level of evidence of CP genetic findings through the process of gene discovery, laboratory study, and finally, clinical application (Table 1). We hope that these proposed criteria will facilitate ongoing dialogue in the field amongst clinicians, basic scientists, and genomic researchers.

**Table 1:** Proposed criteria for prioritizing potential CP-associated genes.

		**Clinical features**	**Variant**	**Gene**
	**Gene discovery**	Clinical phenotype is consistent with CP^*^ (i.e., no progression, consistent movement disorder, early-onset).	1. Not widely represented in population (<0.001% MAF) 2. Inheritance pattern and family history consistent with segregation in family 3. Bioinformatic tools predict deleterious effect on protein function	1. Expressed in nervous system during development or regulates process known to contribute to CP (e.g., inflammation) 2. Mis_Z or pLI score indicates gene intolerance to random variation
**←Increasing levels of confidence**	**Research characterization** Criteria met for gene discovery plus:	1. Similar phenotype (non-progressive, consistent movement disorder with early onset) in same gene identified in matching service (i.e., Genematcher).	1. Loss, gain, or change of function effect on protein verified in laboratory tests OR known LOF variant type (i.e., stop gain, frame shift) 2. Phenotype in patient tissues consistent with change in gene function 3. Statistical enrichment of predicted deleterious variants in this gene in cases compared to controls	1. Similar phenotype in a model organism with gene LOF 2. Present in protein complex with known disease-associated gene(s) 3. Shares molecular pathway with other CP-associated genes
	**Clinical genetics** Criteria met for gene discovery and research characterization plus:	Previous reports of the same gene (recurrence), with: 1. Motor symptoms consistent with CP or neurodevelopmental disorder (motor features unknown) 2. Patient findings (dysmorphic features, organ system involvement, etc.) consistent with previously reported findings	1. Same variant previously reported with similar symptoms 2. Variant in same functional domain as other disease-causing variants 3. Predicted pathogenic/likely pathogenic	1. Documented as a CP-associated gene in OMIM or included in clinical gene panel

*Criteria indicate supportive evidence that support advancement to the next level of confidence. Criteria for each category and type of study ordered from most confidence to least confidence*.

*CP, cerebral palsy; HSP, hereditary spastic paraplegia; LOF, loss of function; MAF, minor allele frequency; NDD, neurodevelopmental disorder. pLI estimates the probability of a gene being loss of function intolerant*.

* *Rosenbaum et al. (1) and Smithers-Sheedy et al. (37)*.

*Adapted from the ACMG guidelines for evaluating variant pathogenicity in Richards et al. (38) taking into account steps and evidence in the process of confirming CP-association starting from gene discovery*.

We have also reviewed the extant literature for cohort-based next-generation sequencing of CP. Our search strategies were expanded to include patients from cohorts of pediatric movement disorders with non-progressive spasticity, dystonia, ataxia, or chorea that meet CP diagnostic criteria (1) with WES sequencing since 2015. We curated 127 gene variants from WES cohort studies and 32 CNVs from array and WES studies (Supplemental Table 1). To determine which genes have strong evidence for causing CP, we identified genes that meet ≥1 criteria from all sections of Table 1. We found 18 genes and 5 CNVs meeting these criteria that have been described in 2 or more patients (Table 2).

**Table 2:** Recurrent CP genes and copy number variants.

**Gene/region**	**# of patients**	**Associated OMIM disorder**	**Primary movement type**	**Citations**
AGAP1	3	–	Spastic dystonic	van Eyk et al. (35), McMichael et al. (10, 12)
AMPD2	2	Spastic paraplegia 63	Spastic	Takezawa et al. (20), Jin et al. (21)
AP4M1	8	Spastic paraplegia 50	Spastic-dystonic diplegia	Jin et al. (21), Jameel et al. (39), Verkerk et al. (40)
ATL1	5	Spastic paraplegia 3	Mostly spastic	Zouvelou et al. (41), Jin et al. (21)
CACNA1A	2	Episodic ataxia, type 2	Ataxic	Zouvelou et al. (41), Takezawa et al. (20)
COL4A1	2	Hemorrhage, intracerebral, susceptibility to	Spastic-dystonic w/generalized hypotonia, myoclonic jerks; ataxia	van Eyk et al. (35)
CTNNB1	5	Neurodevelopmental disorder with spastic diplegia and visual defects	Mostly spastic	Jin et al. (21), Cordeiro et al. (42)
FBXO31	2	Mental retardation, autosomal recessive 45	Spastic	Jin et al. (21)
ITPR1	2	Spinocerebellar ataxia 29, congenital non-progressive	Ataxic	Schnekenberg et al. (19)
KDM7A	2	–	Spastic dystonic	van Eyk et al. (35)
KIF1A	2	Spastic paraplegia 30	Spastic dystonic	van Eyk et al. (35)
MAOB	3	–	Spastic	van Eyk et al. (35)
NT5C2	3	Spastic paraplegia 45	Spastic diplegia	van Eyk et al. (35), Jin et al. (21), Naseer et al. (43)
RHOB	2	–	Spastic dystonic	Jin et al. (21)
SCN2A	2	Episodic ataxia, type 9	Spastic, ataxia, hyperkinesia	Cordeiro et al. (42), Takezawa et al. (20)
SPAST	7	Spastic paraplegia 4	mostly spastic	Takezawa et al. (20), Jin et al. (21), Zouvelou et al. (41)
STXBP1	3	Epileptic encephalopathy, early infantile, 4	Ataxic	Cordeiro et al. (42), Takezawa et al. (20)
TUBA1A	3	Lissencephaly 3	Spastic	Jin et al. (21)
dup Xp22.33	2	–	Hemiplegic	Zarrei et al. (15)
Del Xp22.33	2	–	Spastic, choreoathetotic	Oskoui et al. (11, 14), Corbett et al. (16)
Dup 2p25.3 (MYT1L)	2	MRD39	Spastic, choreoathetotic	Oskoui et al. (11, 14)
del 9p24.3 (KANK1)	2	Cerebral palsy, spastic quadriplegic, 2	Spastic	Oskoui et al. (11, 14), Segel et al. (13)
del 22q11.21	2	22q11.2 deletion syndrome		

*Variants curated from literature of cohort-based WES and filtered to only include genes with at least one high-confidence variant meeting 1 or more criteria from each column in Table 1*.

Identifying genes responsible for “pure” movement disorder phenotypes has proved quite challenging, but themes are emerging. Genes causing the canonically progressive hereditary spastic paraplegia and the early onset, stable course of spastic CP overlap. Several of these genes, including *ATL1, SPAST*, and *AP4* complex members, converge on intracellular membrane trafficking and distribution, regulating the shape of organelles such as the endoplasmic reticulum (44). Genes causing mixed spastic-dystonic CP include *AGAP1, CTNNB1, FBXO31, KDM7A, KIF1A*, and *RHOB*, which indicates diverse biological processes can contribute to motor dysfunction. Genes crucial for basal ganglia development, *NKX2*-1, and cyclic nucleotide regulation in the striatum, including *GNAO1, ADCY5*, and *PDE10A* (45) have been identified in choreic CP. Finally, mutations in several ion channel genes have been found in ataxic CP patients including *KCNC3, ITPR1* (19) and *CACNA1A* (20, 41). Neurotransmission might be more broadly involved as mutations in *STXBP1*, a regulator of syntaxin, have also been found in ataxic CP (19, 20). Taken together, some CP motor subtypes and other motor disorders appear to share not only overlapping phenotypic features, but also overlapping genes and genetic pathways (46).

## CP Gene Functions and Pathways

Several case-control studies have used high-throughput-omics approaches to discover dysfunctional pathways and networks associated with CP. In other areas of disease research integrated-omics approaches have been used to consolidate genomic, epigenomic, and transcriptomic findings, but this approach has yet to be systematically applied to CP. Nonetheless, early findings are proving interesting.

### Genomics

Genes with mutations detected in CP cluster into pathways governing neurite extension including the extracellular matrix, cell-matrix interactions, cytoskeletal dynamics, and Rho GTPase function. Several genes in these pathways cause locomotor impairments in Drosophila loss of function models (21), indicating that CP genes may regulate connectivity of central nervous system circuits regulating movement.

The concept that CP-associated genes regulate nervous system connectivity is further supported by the clinical and genetic overlap of CP with other neurodevelopmental disorders. Patients with CP often have intellectual disability (~45%), epilepsy (~40%), and autism (~7%) (47, 48). Jin et al. (21) found a 1.7–2.0-fold enrichment of NDD genes among CP candidate genes detected by WES. Genetic pleiotropy has been described for several CP candidate genes. For example, *SCN8A* encodes a sodium channel with well-characterized mutations causing epileptic encephalopathies (49). Additionally, *de novo* mutations in *SCN8A* have been identified in CP (10) and intellectual disability without seizures (50). *KCNMA1* encodes a voltage and calcium gated potassium channel with different mutations associated with a spectrum of neurodevelopmental phenotypes including intellectual disability, developmental delay, axial and ataxic hypotonia, epilepsy, and dyskinesia (51, 52). The association of CP with known NDD genes deserves special mention as potential phenotypic expansions as in some cases, movement disorders have not been well-described previously (53). In most cases, the literature has not indicated the absence of a movement disorder but rather has remained silent on the issue, focusing instead on dysmorphic features, intellectual disability, etc. Thus, genetic disruption of brain development can result in CP, other neurodevelopmental disorders, or a combination.

### Epigenomics

Epigenomic analyses reveal alterations in axon guidance, actin cytoskeleton, and cell signaling with parallels to genomic findings. In monozygotic twins discordant for CP, pathway enrichment analysis for alterations in genome-wide DNA methylation at birth (54) shows changes in MAPK signaling, hypoxia-associated signaling, inflammation, cell adhesion, cytokine–cytokine receptor interaction, and Ras signaling. In unrelated individuals with CP, methylation differences have been identified in genes involved in axonal guidance, the actin cytoskeleton, insulin and ephrin receptors, crosstalk between dendritic cells and natural killer cells, TGF-β, Wnt, neuregulin, PI3K/AKT, and tight junction signaling (55). These findings support the notion that perinatal hypoxic-ischemia and inflammatory responses are associated with an eventual CP outcome. However, it is not clear whether a causal relationship exists, as these studies did not control for environmental risk factors, which could themselves account for the epigenetic changes. Additional cohorts studying many more epigenomes with careful attention to age, risk factors, and statistical methods are needed to determine if the changes are consistent, and how that relates to patient outcomes. Further studies linking genomic and epigenomic findings are also warranted.

### Transcriptomics

Transcriptomic analysis of lymphoblastoid cell lines derived from 182 CP patients with a mix of environmental, genetic, and indeterminate etiologies revealed 387 significantly differentially expressed genes (56). Pathway enrichment analysis using this gene set demonstrated downregulated signal transduction and cell signaling pathways including brain-derived neurotrophic factor, upregulated immune function genes, and altered amyloid precursor protein A (APP) processing. Despite differing etiologies and age at the time of collection, the authors found overlap in dysregulation of MAPK signaling that aligned with findings from epigenetic studies. Many of the patients in this cohort have been reported in McMichael et al. (10) or Jin et al. (21) suggesting defects in genes that govern cell signaling (MAPK/PI3K/AKT) may lead to neuronal wiring defects and CP. One fascinating question regarding these findings is whether the observed difference in expression is primary (i.e. fundamentally related to the cause of CP) or secondary (i.e. a consequence of chronic spasticity/dystonia, muscle contracture, etc.) in nature.

Given these findings converge on neurotrophic and stress-response signaling pathways as well as cell adhesion, cytoskeletal maintenance, and actin dynamics, it will be important to replicate and further integrate these results. In the future, stratification of findings by etiology may reveal either consistent or disparate mechanisms. For both epigenetic and transcriptomic studies, the stage of development, time post-injury, and tissue type sampled will impact the resulting profiles. Harmonized study designs across—omics platforms will facilitate cross comparisons between epigenomics, transcriptomics, and proteomics, among other techniques. A serial study of individuals assessed using multiple techniques might be most revealing. Detailed catalogs of patient phenotypes will further facilitate interpretation of these complex findings and may help discern pathways which drives pathophysiology vs. those representing compensatory mechanisms.

## Genetics and The Differential Diagnosis of CP

The international consensus definition for CP defines the disorder as “a group of permanent disorders of the development of movement and posture, causing activity limitation, that are attributed to non-progressive disturbances that occurred in the developing fetal or infant brain” (1). In 2019 the International Cerebral Palsy Genomics Consortium (ICPGC) released a consensus statement reaffirming that CP is defined by clinical phenotype rather than etiology (57). Therefore, a CP diagnosis applies if the definitional criteria are met, regardless if there is a genetic etiology (37). However, a recent survey of physicians who treat people with CP across different specialties revealed variability in current clinical practice. When presented with a hypothetical scenario, only 67% would make a diagnosis of CP if there was a consistent clinical CP phenotype with a genetic cause identified (58). There is thus a need for more clinician awareness and training on genetic etiologies of CP and implications for clinical practice in order to avoid revisionist diagnosis for children who fit clinical criteria for CP.

It is also important for clinicians to be able to distinguish between CP and other disorders to plan treatment and care. Inborn errors of metabolism (IEM) appear early in life with different types of disordered movements, including hypotonia, dystonia, and chorea (59). The stakes are high: 67 of the 110 inborn errors of metabolism that can resemble CP are treatable (59). Therefore, there is a need to quickly and accurately identify the underlying etiology. We posit that CP is not currently, and should not be defined as an “untreatable” disorder. This begs the question: When is it CP and when is it an inborn error of metabolism?

We suggest considering IEM as a potential etiology of CP and using the following classifications based on whether the condition is stable or degenerative:

1. If after treating the metabolic disturbance, the disability resolves without permanent brain injury, the patient should be described as having a treatable IEM.
2. If after treating the metabolic disturbance, the injury to the brain and disability remain but does not worsen, the patient should be described as having CP with a neurometabolic etiology.
3. If the disability progresses, with or without treatment, the patient can be described as having a non-CP rare pediatric movement disorder caused by a degenerative IEM.

A diagnostic classification of both CP and an IEM would be similar to indicating that a person has epilepsy due to an underlying neurometabolic disorder. In many cases, this would be beneficial for access to treatments and services needed for the optimal patient care (37).

Genetic findings previously thought to singularly lead to neurodegenerative disorders have now been identified to lead to non-degenerative motor disorders that meet CP criteria. There is increasing evidence that different mutations in the same gene can lead to either degenerative or developmental phenotypes that probably reflect different effects on cellular biology. Careful phenotyping and follow up of patients with *SPAST* and *ATL1* mutations have revealed that some have phenotypes that are stable over decades (60, 61). *KCNC3* variants from infant-onset disease change channel gating properties and increase neuron excitability compared to variants from adult-onset disease, which were associated with reduced channel activity and high-frequency neuronal firing. Further, these effects can manifest with different temporal patterns, with the infant-associated variant disrupting dendrite and axon branching as a developmental feature absent from the adult-associated variant (62). Mutations in *TRIO* were recently shown to lead to distinct, domain-specific effects on development (63). This evidence suggests that manifestations may vary depending on the nature of a given mutation and potentially other moderating factors. A finding in a gene linked to a potentially progressive disorder should trigger a careful re-appraisal of the patient's phenotype and may influence testing, monitoring, and follow up, but should not reflexively prompt a revision of diagnosis unless clinically appropriate based on the patient's course.

## Are New Genetic Discoveries Changing the Treatment of CP?

New genetic findings are raising new questions. For instance, do genetic mutations disrupt brain development in consistent ways? What imaging findings indicate a genetic cause of CP? There have been several studies on brain malformations in movement disorders (64). Periventricular leukomalacia (PVL) detected by MRI has classically been interpreted as caused by perinatal stress (65). However, PVL was also detected in patients with *ATL1* and *RHOB* variants (21) and 5/12 patients with a neuroimaging finding of PVL had no pre- or perinatal risk factors or complications (66). This finding suggests that rather than representing a single etiology, PVL reflects white matter abnormalities where the timing can be either pre- or perinatal (67). In addition, individuals with CP often have structurally normal or mild, non-specific anatomic MRI findings (68). Functional MRI may add additional dimensionality to the understanding of brain connectivity (69). Compared to those with abnormal MRI findings, CP patients with normal MRIs are more likely to have been born at term and experienced an uneventful perinatal period. They are also more likely to have dyskinetic and diplegic CP types but show no differences in the severity of impairment or presence of comorbidities (70). More study is currently needed to determine how MRI could predict genetic contributions to CP.

Genetic findings can and will inform personalized medicine over time. For instance, in a randomized case-control clinical trial, polymorphisms in immune genes (*IL1*β, *PAI1, IL6R*) were associated with improved neurodevelopmental outcomes, including reduced rates of CP, after magnesium sulfate intervention in women at imminent risk of premature delivery (71). Genetic etiologies may also inform interventions such as deep brain stimulation (DBS). In some cases, DBS outcomes can be anticipated based on prior genetic findings (72, 73). Other genes may inform preventative or monitoring efforts to ensure optimal outcomes, such as avoiding head injury and managing cardiovascular risk factors in *COL4A1* patients. Due to increasingly compelling evidence that CP can have a genetic component, future studies should test whether using WES and CNV analysis can guide clinical care for CP. Surveys of patients and their families with disorders of unknown etiology support the use of WES as part of the diagnostic process, with the greatest benefit reported from those who obtained positive findings (74). This underscores the need for additional gene discovery and translational research to improve the interpretation of such tests and establish clinical guidelines for utilization in diagnostic assessment.

## Discussion

In summary, genetic etiology is a notable contributor to the development of CP, potentially through both disrupted brain development and dysregulated responses to risk factors. A small number of recurrent genes with strong evidence of pathogenicity have been described. However, there are likely hundreds more genes that await discovery and/or validation with large-cohort sequencing and follow up studies on the molecular consequences of patient-associated variants. Future studies will also be needed to provide a more in-depth analysis of the function of genes in individuals with overlapping phenotypes using platforms like Genematcher (36). A comprehensive list of all genes that have been identified for CP, not just those from cohort WES studies reviewed here, will be an essential development for the field, particularly as clinical CP sequencing panels are already being offered. Since CP-associated genes have not been systematically curated in OMIM, this represents an opportunity for the field. In the interim, the inclusion of NDD genes with or without previously described movement disorder phenotypes compatible with CP can serve as a surrogate.

Efforts to find genetic etiologies by CP motor type have been limited, in part due to genetic and phenotypic heterogeneity. Identifying CP-subtypes may become more feasible with meta-analysis with high numbers of patients with detailed clinical information provided for every individual in the cohort, facilitated by the International Cerebral Palsy Genomics Consortium (www.icpgc.org). Identifying genes that can cause specific motor types will be crucial for studying the mechanisms underlying those disorders as well as identifying pathways and other features to guide further gene discovery and the development of personalized treatments.

Progress in understanding CP pathology and developing new treatments has been impeded by the limited availability of animal models for functional studies. As monogenic forms of CP are increasingly identified, this will allow development of important genetic models to better understand changes to neurobiology and development in CP. Studies to date have identified relatively few genes as sequenced cohorts have been comparatively small, compounded by a relative lack of deep phenotyping. As more genetic studies are conducted, there will be an increased need for validation analyses to definitively link variants and genes with cerebral palsy. This improved characterization of individual genes will also facilitate subsequent gene discovery, pathways contributing to CP pathogenesis, and the development of targeted interventions. A better understanding of CP genomics will also enable the identification of risk genes contributing through a multifactorial, rather than strictly monogenic way. Together, the discovery of genetic factors relevant to CP provides new opportunities for detailed study driving development of treatments and interventions to improve the lives of people living with CP. Updating diagnostic criteria and practice parameters to incorporate indications for genetic testing and interpretation should be considered to provide clarity and guidance of how to classify CP considering the evolving genetic landscape.

## Author Contributions

SL conceptualized and wrote the manuscript. SS, BW, and AH contributed to literature review, variant curation, and drafting of the manuscript. SJ, HS-S, MF, and MK made intellectual contributions and edits. All authors approved the final manuscript.

## Conflict of Interest

MK serves as a consultant to PTC Therapeutics. The remaining authors declare that the research was conducted in the absence of any commercial or financial relationships that could be construed as a potential conflict of interest.

## Supplementary Material

Supplemental Table 1 Curated sequence variants from whole exome sequencing studies of cerebral palsy cohorts.

Supplemental Table 2 Curated copy number variants from whole exome and microarray studies of cerebral palsy cohorts.
